# Long-term surgical outcomes of primary retropupillary iris claw intraocular lens implantation for the treatment of intraocular lens dislocation

**DOI:** 10.1038/s41598-020-80292-3

**Published:** 2021-01-12

**Authors:** Eun Young Choi, Chul Hee Lee, Hyun Goo Kang, Jae Yong Han, Suk Ho Byeon, Sung Soo Kim, Hyoung Jun Koh, Min Kim

**Affiliations:** 1 Department of Ophthalmology, Institute of Vision Research, Severance Eye Hospital, Yonsei University College of Medicine, 50-1, Yonseiro, Seodaemun-gu, Seoul, 03722 Korea; 2 Department of Ophthalmology, Institute of Vision Research, Gangnam Severance Hospital, Yonsei University College of Medicine, 211, Eonjuro, Gangnam-gu, Seoul, 06273 Korea

**Keywords:** Lens diseases, Outcomes research

## Abstract

We aimed to investigate the efficacy and safety of primary retropupillary iris claw intraocular lens (R-IOL) implantation in patients with complete intraocular lens (IOL) dislocation. In this single-center retrospective case series, we reviewed the medical records of patients who underwent R-IOL implantation surgery with pars plana vitrectomy for the treatment of IOL dislocation between September 2014 and July 2019. The primary outcome was change in visual acuity (VA) up to 24 months postoperatively. The secondary outcomes included changes in intraocular pressure (IOP), refractive errors, and endothelial cell count (ECC) over the same period. Data of 103 eyes (98 patients) were analyzed. The mean uncorrected VA was significantly improved at one month postoperatively (− 0.69 logMAR, *P* < 0.001), compared to the preoperative value. IOP (− 2.3 mmHg, *P* = 0.008) and ECC (− 333.4 cells/mm^2^, *P* = 0.027) significantly decreased one month post-surgery and remained stable thereafter. Postoperative mean spherical equivalents were similar to the prediction error throughout the follow-up period. IOP elevation (n = 8, 7.8%), cystoid macular edema (n = 4, 3.9%), and dislocation of the R-IOL (n = 10, 9.7%) were managed successfully. Overall, primary R-IOL implantation with pars plana vitrectomy is effective and safe for correcting IOL dislocation due to various causes.

## Introduction

The widespread use of phacoemulsification, which strongly increases the proficiency of cataract operations, has steadily increased over the past 32 years^1^. Consequently, the incidence of intraocular lens (IOL) dislocation has increased with the increasing number of cataract surgeries that were performed. Therefore, the treatment for IOL dislocation is an emerging surgical dilemma and may impose a relatively large public health care burden in the future. In particular, complete IOL dislocation may predispose patients to serious retinal complications, such as vitreous hemorrhage and retinal detachment^2^. In general, surgical management of complete IOL dislocation involves either pars plana vitrectomy and removal of the dislocated IOL, followed by secondary IOL implantation at the surgeon's discretion, or re-fixation of the dislocated IOL without IOL removal.

Many clinicians have tried to develop an effective secondary IOL implantation technique that would yield the best visual outcome with minimal complications. Conventional secondary IOL implantation via scleral sutures has been the treatment of choice, as the position of the scleral-fixated IOL (SF-IOL) is ideal with respect to the anatomy of the eye. However, this technique requires the use of sutures and sufficient operator experience, and has a relatively long operation time^3^. Secondary IOL scleral fixation may lead to other severe intraocular complications, such as vitreous hemorrhage, retinal detachment, choroidal detachment, and suture-related complications^4^. An alternative procedure, secondary IOL implantation of anterior chamber IOLs, was popularized in the 1980s due to its simplicity and efficacy. However, a high risk of developing postoperative secondary glaucoma, uveitis, and endothelial cell decompensation has been reported in cases of anterior chamber IOL^5^ with a particularly inflexible closed-loop design^6^.

Recently, the retropupillary iris claw intraocular lens (R-IOL) has emerged as a viable option for secondary IOL implantation. In the 1980s, Amar first proposed the fixation of an iris claw lens at the posterior surface of the iris^7^. This R-IOL implantation technique gained popularity after Mohr et al. reported the 1-year clinical outcomes for this technique in 2002^8^. Structurally, R-IOL provides better stability and lower risks of IOL tilt or dislocation than iris- or scleral-sutured lenses. In addition, the absence of sutures for IOL scleral fixation lowers the risk for suture-related complications, such as conjunctival erosion, scleromalacia, and endophthalmitis^9,10^. Many previous studies have shown that secondary R-IOL is a safe and efficacious technique for correcting various aphakic conditions with^11–17^ or without IOL dislocation^3,18^. However, the long-term safety and efficacy of simultaneous primary R-IOL implantation and vitrectomy in patients with complete IOL dislocation have not been previously assessed.

Herein, we present our surgical method for correcting IOL dislocation using R-IOL and investigate the long-term efficacy and safety of primary R-IOL implantation in patients with complete IOL dislocation.

## Materials and methods

This was a single-center, retrospective case series study. We reviewed the records and operative reports of all patients who underwent R-IOL implantation (performed by a single surgeon—M. Kim) at the Department of Ophthalmology, Gangnam Severance Hospital, between September 2014 and July 2019. The study was conducted in accordance with the Declaration of Helsinki, and the protocol was approved by the Institutional Review Board at Gangnam Severance Hospital (IRB approval number: 3-2020-0213). The requirement for informed patient consent was waived because the data were anonymized before the analysis.

We have selectively included cases of IOL dislocation involving total dislocation of the in-the-bag IOL into the posterior segment or cases of recurrent complete dislocation of the SF-IOL, which were treated by the removal of the dislocated IOL followed by R-IOL implantation. Cases in which dislocated IOLs were rescued and repositioned were not included in this study. We included all patients in the analysis, including those with various complicated retinal pathologies.

### Preoperative examination

All past medical history and preoperative ophthalmologic data for each patient were reviewed. Preoperative uncorrected visual acuity (VA) and best-corrected VA (BCVA) obtained using the Snellen vision chart, which were converted to logMAR values for statistical analysis, were obtained. Preoperative intraocular pressure (IOP) was measured using a non-contact tonometer. Preoperative refractive data were obtained using an auto kerato-refractometer (KR-1, Topcon Medical Systems, Inc.; Oakland, NJ, USA); spherical equivalent (SE) and corneal astigmatism were also calculated. The auto KR values were used as the starting point to perform manifest refraction, and the best subjective correction for a distance of 40 cm was found after adjustment of spherical power, as well as the cylinder axis and power, by ± 0.25 D in 1° increments. Biometry measurements obtained using a ZEISS IOLMaster 500 (Carl Zeiss AG; Heidenheim, Germany) were reviewed and the ‘prediction error’, which is the predicted target refraction before surgery, was recorded. IOL calculations were always performed with the SRK/T formula using an A-constant of 116.9, which is the manufacturer’s recommendation for retropupillary fixation. This constant was used in the laser interferometry biometry mode. Finally, endothelial cell count (ECC) performed automatically using specular microscopy (CellChek XL, Konan Medical USA Inc.; Irvine, CA, USA) was analyzed.

### Surgical technique

**Figure 1:**
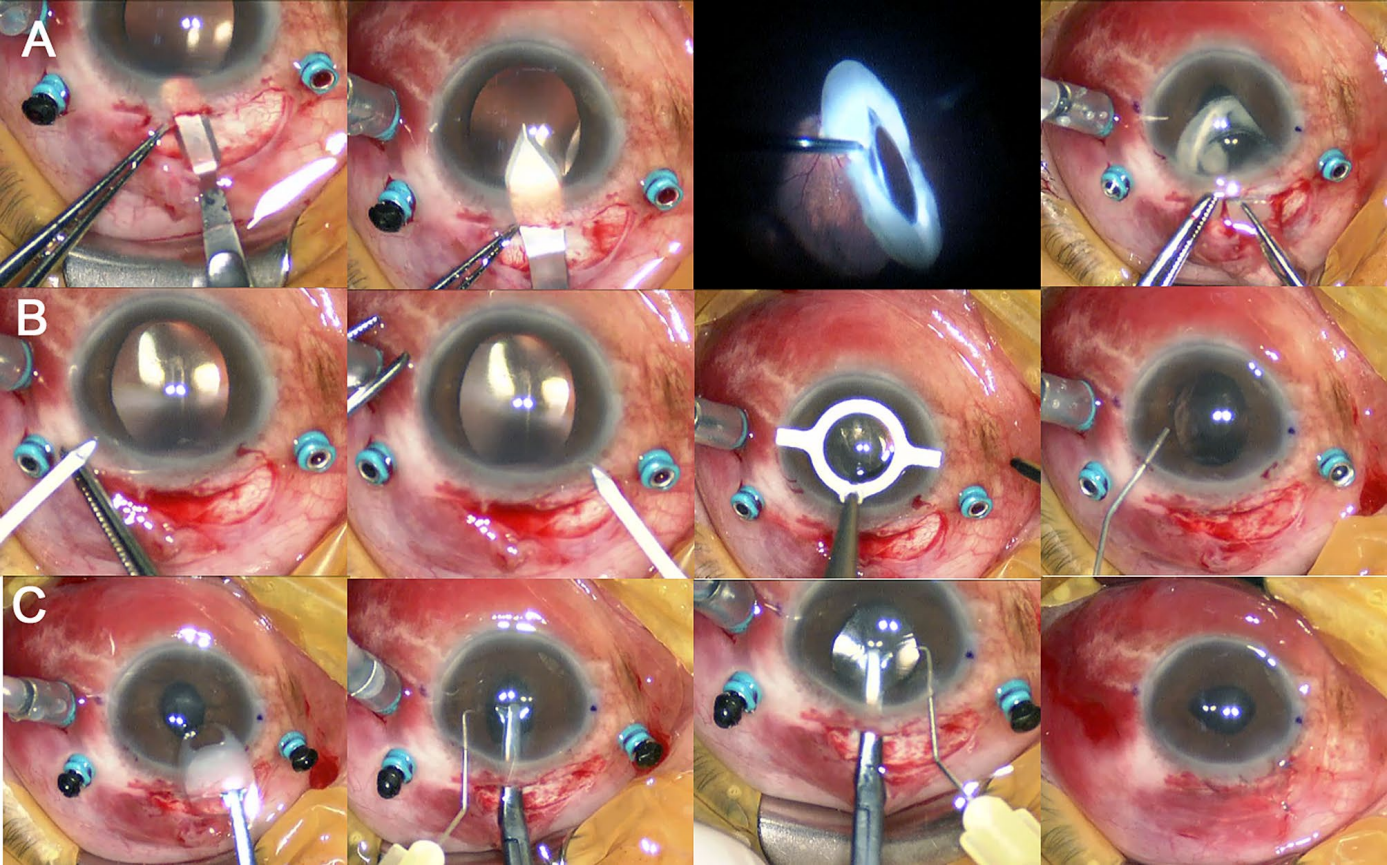
Intraoperative photographs showing the surgical procedures for retropupillary iris claw intraocular lens (R-IOL) implantation. An 80-year-old male patient underwent R-IOL implantation in the left eye for the treatment of intraocular lens (IOL) dislocation under local anesthesia. A 25-gauge trans pars plana vitrectomy was performed. After performing the core vitrectomy and peripheral shaving, (**A**) a 5.5 mm scleral flap was made using a round blade under the conjunctival flap. A scleral tunnel incision was made using a steel keratome. After grasping the posteriorly dislocated capsular bag-IOL complex using a 25-gauge vitrectomy probe, the dislocated capsular bag-IOL complex was translocated into the anterior chamber and gently removed through the scleral tunnel using IOL forceps. (**B**) Two paracentesis incisions were made at the 2 o'clock and 10 o'clock positions using a sharp blade. The horizontal plane indicating the IOL haptic enclavation sites was marked using a marking pen. Viscoelastic material was injected into the anterior chamber through the scleral incision site. (**C**) The R-IOL was prepared for implantation and inserted into the anterior chamber through the previously formed scleral tunnel using ARTISAN forceps and was rotated. The haptics of the R-IOL were fixated at the 3 o'clock and 9 o'clock positions of the iris using an enclavation needle. The remaining viscoelastic material was removed, and the scleral tunnel incision was closed using 10-0 nylon sutures. The superior conjunctival flap was closed using 7-0 vicryl sutures.

A 25-gauge pars plana vitrectomy was performed (CONSTELLATION Vision System, Alcon; Fort Worth, TX, USA) for each patient. After the core vitrectomy and peripheral shaving to free the dislocated IOL from the vitreous, the anterior chamber was filled with dispersive viscoelastics (VISCOAT, Alcon; Fort Worth, TX, USA), and the dislocated IOL complex was lifted up into the anterior chamber. Then, a conjunctival incision was made from the 11 o’clock position to the 1 o’clock position using spring scissors. A 5.5 mm scleral flap was created using a crescent blade and a sclerocorneal incision was made using a steel keratome. The dislocated IOL was gently removed through the scleral tunnel. Two paracentesis incisions were made at the 2 o’clock and 10 o’clock positions using a sharp blade, and the R-IOL (ARTISAN Aphakia model 205, OPHTEC BV; Groningen, Netherlands) was prepared for implantation. After filling the anterior chamber with viscoelastic material, the R-IOL was inserted through the previously formed scleral tunnel using ARTISAN forceps (OPHTEC BV; Groningen, Netherlands). The claws of the R-IOL were fixated at the 3 o’clock and 9 o’clock positions of the posterior iris using an enclavation needle (OPHTEC BV; Groningen, Netherlands). After closing the scleral tunnel incision using 10-0 nylon sutures, the remaining viscoelastic material was removed by automatic irrigation and aspiration. The superior conjunctival incision was closed using 7-0 vicryl sutures. Figure 1 presents a detailed description of the surgical technique.

### Postoperative examination

All postoperative data (uncorrected VA, BCVA, IOP, refractive errors, and ECC) were acquired at 1, 6, 12, and 24 months postoperatively. The SE outcome after surgery was described as the ‘postoperative refraction’. If there were any missing values in the postoperative data, the subjects were excluded from the analysis. Postoperative complications and corresponding treatments were documented for each case.

### Statistical analyses

All statistical analyses were performed using Statistical package for the social sciences (SPSS/IBM Corporation; Chicago, IL, USA) version 23.0 software for Windows. The Shapiro–Wilk test was used to examine normality. A repeated measures analysis of variance (RM-ANOVA) with a post-hoc Fisher’s least significant difference (LSD) test was performed to analyze the differences in variables between multiple time-points. A paired *t*-test was used to compare two time-points. *P* values < 0.05 were considered to be statistically significant. Data are presented as the mean ± standard deviation, unless otherwise noted.

## Results

### Preoperative baseline characteristics

Among the 134 patients who underwent R-IOL implantation surgery, 29 patients who underwent R-IOL implantation for other causes (aphakia correction, n = 12; posterior capsular rupture or zonular dialysis during cataract surgery, n = 17) were excluded. Additionally, seven patients were excluded due to loss of follow-up within 24 months postoperatively. Therefore, a total of 103 eyes of 98 patients that met the inclusion criteria were included in the present study.

Table 1 summarizes the preoperative baseline characteristics of patients enrolled in the study. The mean age of the study population was 60.9 ± 12.3 years (range, 18–88 years). The average follow-up duration was 38.2 ± 6.2 months (range, 24–61 months) and the study population was predominantly comprised of men (90.8%). Preoperatively, the mean uncorrected VA was 0.97 ± 0.75 and the mean BCVA was 0.34 ± 0.58 on the logarithm of the minimum angle of resolution (logMAR) scale. The preoperative SE was + 4.96 ± 5.99 diopters (D) (range, − 19 to + 17.75 D), and corneal astigmatism was + 1.12 ± 0.82 D (range, 0 to + 4.88 D). The mean axial length was 25.1 ± 2.3 mm (range, 21.69–34.85 mm). The average IOP was 17.0 ± 7.7 mmHg and the endothelial cell density was 2164.1 ± 686.2 cells/mm^2^. We speculated that IOL dislocation was caused by zonular weakness in 94 eyes (91.3%), which included six eyes with a history of pseudoexfoliation, four eyes had damage to trauma, 24 eyes (23.3%) exhibited high myopia (axial length ≥ 26.5 mm), eight eyes (7.8%) had previously undergone vitreoretinal surgery (e.g., retinal detachment, phakic subluxation, and endophthalmitis), and two eyes (1.9%) had previously received intravitreal injections (e.g., for diabetic retinopathy and retinal vein occlusion). In-the-bag IOL dislocations were noted in 88 eyes (85.4%), whereas out-of-the-bag IOL dislocations were observed in 15 eyes (14.6%). Among the latter 15 eyes, dislocation of previous SF-IOLs was noted in nine cases (8.7%).

**Table 1:** Baseline characteristics of the patients who underwent retropupillary iris claw intraocular lens (IOL) implantation surgery with pars plana vitrectomy for the treatment of an IOL dislocation.

**Total number of patients/ eyes**	98/ 103
Age (years)	60.9 ± 12.3
Men/Women	89 (90.8)/9 (9.2)
Mean follow-up duration (months)	38.2 ± 6.2
**Preoperative measurements**	
Uncorrected visual acuity (logMAR)	0.97 ± 0.75
Best-corrected visual acuity (logMAR)	0.34 ± 0.58
Spherical equivalent (diopter)	4.96 ± 5.99
Corneal astigmatism (diopter)	1.12 ± 0.82
Axial length (mm)	25.1 ± 2.3
Intraocular pressure (mmHg)	17.0 ± 7.7
Endothelial cell count (/mm^2^)	2164.1 ± 686.2
**Speculated cause of IOL dislocation**	
Zonular weakness	94 (91.3)
High myopia (axial length ≥ 26.5 mm)	24 (23.3)
Previous vitreoretinal surgery	8 (7.8)
A history of pseudoexfoliation	6 (6.1)
Post-traumatic	4 (4.0)
Previous intravitreal injections	2 (1.9)
Unknown cause	50 (48.5)
Dislocation of scleral-fixated IOLs	9 (8.7)

logMAR, logarithm of the minimum angle of resolution.

Values are expressed as means ± standard deviations, except where indicated as number (percentage) of subjects.

### Visual acuity

**Figure 2:**
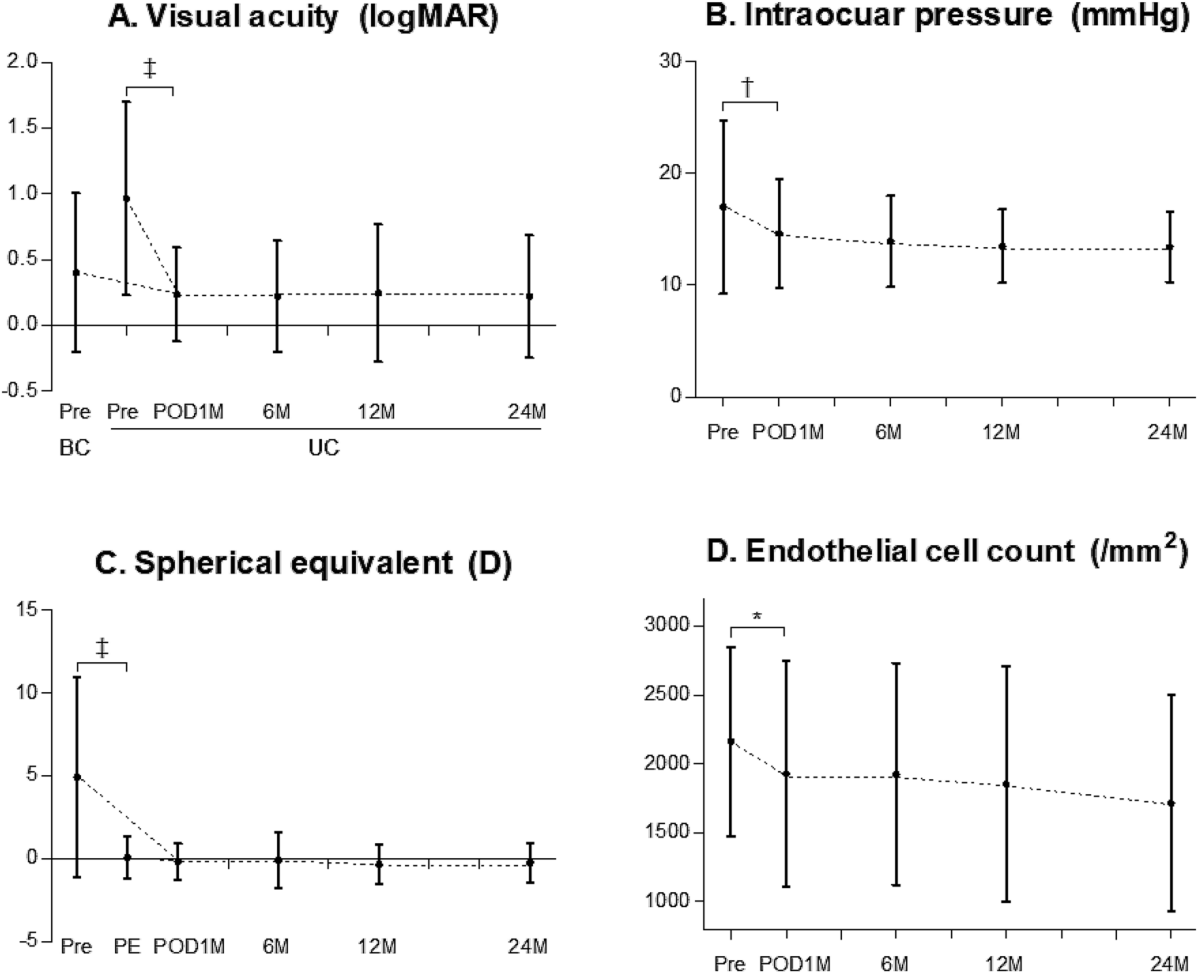
Changes in (**A**) visual acuity, (**B**) intraocular pressure, (**C**) spherical equivalent, and (D) endothelial cell count before and after retropupillary iris claw intraocular lens implantation for the treatment of intraocular lens dislocation. LogMAR, logarithm of the minimum angle of resolution; Pre, preoperative; POD, postoperative days; M, month; BC, best-corrected; UC, uncorrected; D, diopter; PE, prediction error. Repeated measures analysis of variance with post-hoc Fisher’s LSD test for statistical analysis was performed (*P < 0.05, ^†^*P* < 0.01, ^‡^*P* < 0.001). The small black dots represent mean data points and error bars represent standard deviations.

The changes in vision before and after the R-IOL implantation surgery are shown in Fig. 2A. The mean uncorrected VA values 1, 6, 12, and 24 months postoperatively were 0.28 ± 0.44, 0.22 ± 0.46, 0.19 ± 0.45, and 0.22 ± 0.46 logMAR, respectively. The uncorrected VA one month postoperatively showed significant improvement (*P* < 0.001; RM-ANOVA with the Fisher’s LSD method) when compared with the preoperative uncorrected VA (0.97 ± 0.75 logMAR); it was similar to the preoperative BCVA (0.34 ± 0.58 logMAR, *P* = 0.99). The improvement in VA at one month postoperatively did not change significantly during the follow-up period (all *P* values < 0.05). The mean postoperative BCVA at one month after surgery (0.20 ± 0.29 logMAR) was similar to the uncorrected VA measured at the same time point (*P* = 0.91; paired t-test); this indicated a significant improvement when compared with the preoperative BCVA (*P* = 0.013; paired t-test).

### Intraocular pressure

Figure 2B shows the change in IOP before and after R-IOL surgery. The mean IOP values 1, 6, 12, and 24 months after the operation were 14.7 ± 4.9, 14.3 ± 4.2, 14.0 ± 3.5, and 14.1 ± 3.3 mmHg, respectively. The IOP was significantly decreased one month after the surgery (mean difference, − 2.3 mmHg, *P* = 0.008) when compared with the preoperative IOP (17.0 ± 7.7 mmHg); there were no additional significant changes during the follow-up period (*P* values > 0.05; RM-ANOVA with Fisher’s LSD method).

### Refractive error

The changes in SE before and after R-IOL surgery are presented in Fig. 2C. The mean PR values at 1, 6, 12, and 24 months were − 0.41 ± 1.26, − 0.18 ± 2.00, − 0.40 ± 1.29, and − 0.68 ± 1.37 D, respectively. When compared to the preoperative SE (+ 4.96 ± 5.99 D), a significant change was noted only at one month postoperatively (*P* < 0.001; RM-ANOVA with Fisher’s LSD method). There was no significant difference between the mean prediction error (− 0.56 ± 0.98 D) and the 1-month postoperative refraction (*P* = 0.21; paired *t*-test). The 1-month postoperative refraction was within ± 0.5 D, with prediction error observed in 74 eyes (71.8%). There were no additional significant changes in postoperative refraction between one month and 24 months after surgery (all *P* values > 0.05; RM-ANOVA with Fisher’s LSD method). A slight increase in the mean cornea astigmatism was observed at the end of the follow-up period (1.12 ± 0.83 to 1.65 ± 1.23 D, *P* < 0.001; paired *t*-test). According to the slit lamp examination results, there was no apparent IOL tilt or decentration in any patients from our case series.

### Endothelial cell count

The changes in endothelial cell density are shown in Fig. 2D. The mean ECC values 1, 6, 12, and 24 months after the operation were 1676.8 ± 861.7, 1703.7 ± 915.3, 1740.3 ± 907.5, and 1637.8 ± 870.0 cells/mm^2^, respectively. A RM-ANOVA showed an overall decline in ECC over time (*P* = 0.021). A significant reduction in ECC was observed, but only for the period from before the surgery to one month after the surgery (− 16.6%, *P* = 0.027; Fisher’s LSD method).

### Postoperative complications

Complications that developed after the R-IOL implantation surgery are summarized in Table 2. A mild degree of iris atrophy or a focal defect at the enclavation site was noted in 12 eyes (11.7%). Eight eyes (7.8%) had transient IOP elevations. Of these eight eyes, IOP was normalized in six eyes after instillation of anti-glaucoma eye drops for one week. In one patient, IOP elevation was normalized six months after instillation of dorzolamide-timolol and latanoprost. Delayed IOP elevation three months after the operation was noted in another patient who had a history of chronic angle closure and required treatment with dorzolamide-timolol, latanoprost, and oral acetazolamide for one month.

**Table 2:** Postoperative complications of eyes that underwent retropupillary iris claw intraocular lens (IOL) implantation surgery combined with pars plana vitrectomy for the treatment of an IOL dislocation.

Postoperative complications	N (%)
None	71 (68.9)
Iris atrophy/ focal defect	12 (11.7)
Transient elevation of intraocular pressure	8 (7.8)
Disenclavation	10 (9.7)
Cystoid macular edema	4 (3.9)

R-IOL disenclavations were encountered in 10 eyes (9.7%). Disenclavation of the R-IOL occurred significantly more frequently in eyes with iris atrophy (five eyes [41.7%]) than in those without it (five eyes [5.5%]) (*P* = 0.002; Fisher’s exact test). The R-IOL disenclavations occurred shortly after the surgery in three cases; in two cases, the R-IOL disenclavation occurred three months postoperatively, and in one case, it occurred one week postoperatively. Patients with a R-IOL disenclavation required simple re-fixation using enclavation needles. The R-IOLs were stable after the re-fixation and did not require additional manipulation thereafter. There were no significant changes in BCVA, refractive errors, IOP, or ECC before and after R-IOL re-enclavation.

In addition, four patients (3.9%) had cystoid macular edema (CME). The incidence of CME was significantly higher in eyes that had previous re-enclavation (two eyes [20%]) than in those that did not (two eyes [2.2%]) (*P* = 0.046; Fisher’s exact test). CME occurred within six months after the operation in three cases. These cases of early-onset CME were treated with an intravitreal injection of bevacizumab (1.25 mg) in addition to topical nonsteroidal anti-inflammatory drugs. One month after the injection, the CMEs were completely resolved in all cases. At 1-year follow-up, the three patients with early-onset CME had a BCVA that was maintained at 0.1 logMAR, without any further recurrence of CME. Chronic CME recurrence was observed 12 months postoperatively in only one patient who had underlying diabetic retinopathy.

## Discussion

Herein, we investigated the safety and efficacy of primary R-IOL implantation for correcting an IOL dislocation. R-IOL implantation combined with IOL removal and pars plana vitrectomy provided significant improvements in uncorrected VA (average, − 0.69 logMAR) at one month postoperatively as compared to the preoperative values. The VA improvement after R-IOL implantation for an IOL dislocation was well-sustained during the 24 months after the surgery.

Despite concerns of IOP elevation, IOP was not elevated in most cases in our study. In fact, the postoperative mean IOP was significantly lower than the preoperative IOP at the 1-month follow-up postoperatively and remained stable thereafter. In agreement with our results, previous studies demonstrated that the mean IOP at the time of last follow-up after R-IOL implantation was significantly lower than^19^ or similar to the preoperative IOP^16,17^. Furthermore, six of the eight eyes that experienced IOP elevation required just a week of treatment with IOP lowering agents, indicating that the IOP elevation may be due to post-vitrectomy inflammation rather than the R-IOL itself. Baykara et al. showed that the anatomic characteristics of the anterior segment are preserved after R-IOL implantation^20^. This indicates that R-IOL does not induce angle closure or pupillary block under normal circumstances. In addition, there were no cases of peripheral anterior synechiae even 24 months after the surgery, which also indicates that there is a low risk of chronic angle closure after R-IOL implantation.

There was no significant difference between the prediction error and postoperative refraction of the implanted IOL. Subsequently, the patients had very satisfactory visual quality as none of them who underwent this surgery had any postoperative discomfort affecting visual quality. Considerable SE error and, more commonly, IOL tilt that affect visual quality have been observed with SF-IOLs in previous studies^14,21–23^. These complications result in higher-order aberrations and a deterioration of visual quality^24^. In contrast, R-IOL implantation may result in small SE variations (− 0.50 to − 0.94 D) but, it has a low risk of inducing IOL tilt^12,17^, as new needle sclerotomies would not be required for R-IOL. The mean surgically induced astigmatism after a 5.5 mm scleral incision for R-IOL implantation has been shown to be as subtle as + 0.65 D at 171°^25^. However, refraction changes have been observed due to R-IOL shift, depending on the positioning of the head^26^. Further studies comparing the IOL tilt and SE error between eyes that underwent R-IOL implantation and eyes that underwent scleral IOL fixation are required to make definite conclusions.

R-IOL fixation resulted in a 16.6% decline in ECCs at the 1-month follow-up postoperatively. However, there was no further ECC reduction until 24 months after the surgery. Furthermore, no patient developed bullous keratopathy postoperatively. In a recent randomized clinical trial that studied cases with IOL dislocations for six months, an average ECC loss of 10% was observed in patients who underwent IOL replacement with R-IOL, whereas a 3% ECC decompensation was observed in the IOL repositioning group^13^. However, in other studies, R-IOL implantation for correcting aphakia did not significantly decrease ECC, even in patients who had undergone penetrating keratoplasty for bullous keratopathy^27–29^ Recently, the simultaneous performance of R-IOL implantation and Descemet endothelial keratoplasty has been proposed as a safe technique to treat aphakia with corneal decompensation^30,31^. No significant difference in ECC between IC-IOL (anteropupillary) and in-the-bag IOL has been reported^32^. In addition, no significant changes in ECC have been observed in either anterior or posterior iris-claw implantation procedures within five years of follow-up^14^. Stürmer argues that the ECC decline might be what one would expect after surgical IOL exchange and not necessarily from the use of R-IOL^33^. We believe that IOL repositioning and aphakia correction using the R-IOL would result in a smaller risk of endothelial injury because nothing is removed from the intraocular space. Additionally, IOL replacement itself presents a risk for endothelial decompensation since the maneuvers related to the removal of the dislocated IOL through the scleral tunnel may cause some trauma to the corneal endothelium. Furthermore, our study shows that the ECC decline did not significantly progress after one month postoperatively, which may signify that the ECC decline was primarily due to surgical factors related to IOL removal rather than problems of R-IOL itself. The accuracy of ECC could be influenced by variations in cell area and image clarity, as we used an automated measurement technique.

Previous studies have reported high postoperative complication rates related with scleral-fixation of IOLs. Vote et al. reported that 30 of the 61 (49%) SF-IOL eyes required two or more procedures to reverse significant peri- or postoperative complications. The main indication, which accounted for 57% cases requiring reoperations, was caused by breakage of the polypropylene suture^34^. In a study performed in an Asian population, a postoperative complication rate of 24.0% (25 of 104 eyes), mostly suture-related, was reported for SF-IOLs^35^. Although our study group has previously shown that suture-related complication rates decrease when simultaneous IOL rescue with suture-less intrascleral tunnel fixation is performed, this technique is limited in that it can only be used in cases with 3-piece IOL dislocation^36^.

In our study, 9.7% of the eyes required additional procedures for the correction of partially disenclavated R-IOLs during the 2-year follow-up period. Previously reported disenclavation rates of R-IOL have varied between studies. Similar to our results, a 6.6% disenclavation rate of 122 eyes was reported over 7.5 months^17^, whereas Gonnermann et al. reported a higher disenclavation rate (14% of 137 R-IOL eyes) over 6.7 months^19^. In other studies of R-IOL eyes, disenclavation has been observed at a lower rate; for example, one reported disenclavation in 3/320 (0.9%) eyes^12^ and another reported 2/93 (2.1%) eyes being affected^14^ during a 5-year follow-up period. Although there are limitations that prevent direct comparisons between previous reports, the disenclavation rates of R-IOL (0.9–14%) seem to be relatively lower than the dislocation rates of SF-IOL (7.9–25.9%)^3,34,37^. Therefore, a long-term enclavation state of R-IOL might be more stable or sustainable than a conventional scleral fixation state. In our study, the three cases of early disenclavation may indicate insufficient tissue enclavation during the first operation. The difficulty associated with visualization of the posterior surface of the iris during surgery hampers the assessment of the amount of enclaved iris tissue. Simple R-IOL re-fixation using an enclavation needle was sufficient to correct all cases of R-IOL disenclavation. Since complete vitrectomy was performed previously to remove the dislocated IOL, there was no need for additional vitrectomies.

Since a previous study on iris-sutured IOLs reported severe inflammation immediately after the operation^38^, questions have been raised regarding the risk of postoperative inflammation of R-IOL, because it is a surgery that requires iris manipulation. Madhivanan et al. reported that postoperative iritis was more common in R-IOL than in SF-IOL^15^. However, there was no significant difference between the two in other comparative studies^3,14^. In our study, no patient developed significant anterior or posterior inflammation after R-IOL implantation. The reason for the low incidence of inflammation after R-IOL implantation may be due to the difference in the IOL material and the method used to fixate the IOL on the iris. While iris-sutured IOLs are primarily acrylic and require sutures that can injure the blood-aqueous barrier at the endothelium of the iris vessels, R-IOLs (Artisan® Aphakia model 205, Ophtec, Groningen, Netherlands) are suture-less and are composed of polymethyl methacrylate, which induces lesser leukocytic chemotaxis and inflammation than acrylic IOLs^39^. Therefore, R-IOLs may be safer in terms of inflammation than iris-sutured IOLs.

Although the incidence of postoperative CME was low (3.9%) in this study, Gonnermann et al. reported a CME incidence rate of 8.7% (average follow-up duration, 6.7 months). The inclusion of many cases with possible uveal pathologies, such as previous ocular trauma (37.5%), intracapsular cataract extraction (28.1%), and uveitis (6.3%), may have affected the study results of Gonnermann et al.^19^. In addition, they did not perform total vitrectomy for all patients, which also may increase the risk of CME due to vitreomacular traction^19,40^. Recently, a 4.9% incidence of CME was reported for 122 eyes over a 7.5-month follow-up period, including cases in which CME was present preoperatively^17^. In contrast, patients with uveitis were not included in our study population, and all included patients underwent total pars plana vitrectomy. This may explain our low incidence rate of postoperative CME despite a long follow-up duration. Three patients with delayed CME, at an average of 8 months (two cases at 6 months and one case at 12 months), did not have preoperative risk factors, such as diabetes, preoperative prostaglandin use, epiretinal membrane, or retinal vein occlusion^41^. Additionally, they had no pre-existing vitreoretinal pathologies preoperatively. Surgical risk factors, such as vitreous loss, vitreomacular traction, and vitreous traction at incision sites^40^, were precluded by total pars plana vitrectomy. Fluorescein angiography showed a typical petalloid pattern in late-phase frames without signs of secondary causes of CME. We speculated that the three cases of CME were of delayed Irvine-Gass syndrome, which were successfully treated with an intravitreal injection of bevacizumab (1.25 mg)^42–44^ and topical nonsteroidal anti-inflammatory drugs.

Mild iris atrophy or a focal defect at the enclavation site was observed in 12 eyes (11.7%). These patients did not experience related uncomfortable symptoms, such as photophobia, glare, or multiplopia, during the mean follow-up period of 28 months. However, iris atrophy appears to be associated with a higher risk of disenclavation. The mechanism for iris atrophy may be ischemia or mechanical stress on the iris sphincter muscles at the enclavation site. Further studies are required to clarify why iris atrophy occurs after R-IOL implantation, and a longer follow-up duration may be needed to confirm whether cases of iris atrophy are negligible in clinical practice. Pupil ovalization occurs frequently after R-IOL implantation. Gonnermann et al. reported that it occurred in 13.9% of the patients examined, with a mean follow-up duration of five months^19^. Baykara et al. found persistent pupil ovalization after posterior iris claw IOL implantation in 12.7% of eyes examined, with a follow-up duration of nine months^20^. In our study, iris ovalization was observed in 10.7% of the included eyes.

There was a higher percentage of male than female patients who underwent R-IOL implantation surgery for the treatment of IOL dislocation, which is consistent with previous studies^45–47^. Men are more prone to ocular trauma that might have occurred decades ago, which can be ignored and left untreated until the time of cataract surgery. In addition, several causes of zonule weakness are known to be predominant in men, including intraoperative floppy iris syndrome associated with the use of alpha-adrenergic receptor antagonists for treating benign prostate hyperplasia^48^ and connective tissue disorders, such as Marfan syndrome^49^.

The limitations of this study include its retrospective design, the heterogeneous ophthalmologic history, and the absence of control groups. It was not possible to conduct a comparative analysis for this study because of lack of sufficient data based on other methods of IOL scleral fixation. There have, however, been a few randomized trials that have reported comparable outcomes for SF-IOL and R-IOL techniques; for example, one study assessed 30 cases of pediatric aphakia following the removal of congenital cataracts^3^ and another assessed 85 cases with partially dislocated in-the-bag IOL^13^. Further studies are therefore required to compare the results of primary R-IOL implantation with those of other IOL implantation methods in patients with IOL dislocation, with a focus on the stability of IOLs and the associated complications. In addition, more long-term outcomes should be investigated in terms of postoperative chronic inflammation and ECC reduction especially in cases of re-enclavation after primary R-IOL implantation.

To the best of our knowledge, this is the largest study on the efficacy and safety of primary R-IOL implantation solely for the treatment of IOL dislocation. We report visual outcomes and complications that are comparable to or better than those reported for scleral-fixated IOLs. Although there is no consensus on the best surgical technique for the treatment of IOL dislocation, we believe that R-IOL implantation combined with pars plana vitrectomy and the removal of the dislocated IOL is an effective and safe surgical option for the correction of IOL dislocation due to various causes.
